# Diagnostic performance of lactate dehydrogenase as a potential biomarker in predicting preeclampsia and associated factors

**DOI:** 10.3389/fmed.2024.1240848

**Published:** 2024-05-10

**Authors:** Awgichew Behaile Teklemariam, Endeshaw Chekol Abebe, Melaku Mekonnen Agidew, Atalo Agemas Ayenew, Misganaw Asmamaw Mengistie, Nega Dagnew Baye, Zelalem Tilahun Muche

**Affiliations:** ^1^Department of Medical Biochemistry, College of Health Sciences, Debre Tabor University, Debre Tabor, Ethiopia; ^2^Department of Human Anatomy, College of Health Sciences, Debre Tabor University, Debre Tabor, Ethiopia; ^3^Department of Medical Physiology, College of Health Sciences, Debre Tabor University, Debre Tabor, Ethiopia

**Keywords:** preeclampsia, LDH, DBCSH, AUC, ROC

## Abstract

**Background:**

Preeclampsia (PE), a pregnancy specific syndrome, is defined as new-onset hypertension (≥140/90 mmHg) and proteinuria diagnosed after gestational week 20 or new-onset pre-eclampsia associated signs in the absence of proteinuria, and it may tend to present as late as 4–6 weeks’ postpartum period. It is a leading cause of maternal mortality in both developed and developing countries. In order to prevent PE, the disease must be diagnosed at its earliest stage, however, the triads of high blood pressure, edema and albuminuria is neither specific nor sensitive enough for diagnosing the disease. Lactate dehydrogenase (LDH) is useful biochemical marker reflecting the occurrence of complications associated with preeclampsia. Besides, it has been suggested as potential biomarker to predict the severity of preeclampsia and as indicator of multi-organ involvement. The aim of this study was to investigate the diagnostic accuracy of LDH, which is affordable and easy to test, as a potential clinical biomarker to predict onset of preeclampsia.

**Methods:**

A hospital based cross-sectional study was conducted as of September 9 to December 24, 2022 at Debre Birhan Comprehensive Specialized Hospital (DBCSH). A total of 132 study subjects (66 preeclamptic and 66 normotensive controls) were enrolled in the study. A receiver operating characteristics (ROC) curve was used to calculate the area under the curve (AUC) and determine diagnostic accuracy of LDH. Youden’s index was used to identify an optimal cut-off point for LDH in detecting preeclampsia associated complications.

**Result:**

AUC for LDH was found to be 0.963 (95% CI, 0.91, 1.0; *p* = 0.000) from ROC curve analysis. An optimal cut-off point for LDH was 376.5 U/L having a sensitivity and specificity of 87.5 and 90.8%, respectively.

**Conclusion:**

Serum LDH had an AUC of greater than 0.8 and showed good diagnostic accuracy in predicting development of preeclampsia. Disease duration, gestational age, systolic and diastolic blood pressure among enormous number of predictor variables had association with serum level of LDH.

## Introduction

Preeclampsia (PE), a pregnancy specific syndrome, is defined as new-onset hypertension (≥140/90 mmHg) and proteinuria diagnosed after gestational week 20 or new-onset pre-eclampsia associated signs in the absence of proteinuria, and it is the leading cause for maternal and perinatal mortality and death worldwide (1–4).

Updated diagnostic criteria for preeclampsia include blood pressure ≥ 140/90 mmHg noted twice within 6 h after 20 weeks of pregnancy in women who were not hypertensive prior to conception or in those who had previous chronic hypertensive disorders, as well as the coexistence of one or more of the following new-onset conditions: spot urine protein/creatinine >30 mg/mmol (0.3 mg/mg) or > 300 mg/day or at least 1 g/L (2^+^ on dipstick testing), other maternal organ dysfunctions, particularly, renal insufficiency (creatinine >90 μmol/L; 1.02 mg/dL), liver involvement (doubling of serum transaminases and/or severe right upper quadrant pain), neurological complications (eclampsia, altered mental status, blindness, stroke, or more frequently hyperreflexia when accompanied by clonus and severe headaches when accompanied by hyperreflexia and persistent visual scotomata), hematological complications (platelet count <150,000/dL, Disseminated Intracoagulopathy, and hemolysis), fetal growth restriction (5).

PE can cause serious acute maternal complications, including eclampsia, stroke, placental abruption, disseminated intravascular coagulation, HELLP (hemolysis, elevated liver enzymes, and low platelets), liver hemorrhage or rupture, pulmonary edema, adult respiratory distress syndrome, acute renal failure, and death (1, 5). PE can complicate up to 5% of all pregnancies. Pre-eclampsia problems are responsible for more than 50,000 maternal fatalities annually worldwide. Maternal mortality rates can reach 15% in developing nations, when inadequate access to maternal care is a serious issue, as opposed to 0–1.8% in affluent nations. Fetal growth restriction (FGR), stillbirth, iatrogenic premature delivery, neonatal problems, and long-term effects are among the perinatal complications of pre-eclampsia (1).

Preeclampsia’s clinical signs overlap with those of other medical disorders seen in pregnancy, such as liver disease, renal disease, chronic hypertension, and idiopathic thrombocytopenic purpura, which makes the diagnosis more challenging. Additionally, the typical clinical diagnostic criteria, such as proteinuria and/or hypertension, are not reliable enough to forecast the disease’s unfavorable prognosis. Making an improved diagnostic tool that is connected to the disease’s pathophysiology is an important goal and a necessary step for developing novel treatment options because preeclampsia is a prominent cause of maternal death in both industrialized and developing nations (6).

Lactate dehydrogenase (LDH) is useful biochemical marker reflecting the occurrence of complications associated with preeclampsia, which are preventable and can be managed adequately if identified at their early stage. It has also been proposed as a possible biomarker for predicting preeclampsia severity and as an indicator of multi-organ involvement (4, 7). It is most commonly used to assess the existence of tissue damage caused by endothelial dysfunction (8, 9). As a result, estimating serum LDH levels in preeclamptic women may be important for optimal patient management in order to reduce maternal and fetal morbidity and mortality (10). Furthermore, serum LDH was revealed to be a good predictor of PE severity and poor fetal outcome (10–12).

LDH is an ubiquitous and intracellular enzyme, which catalyzes the interconversion of lactate and pyruvate, and its elevated level in serum indicates cellular death and leakage of the enzyme from the cell (7). Human LDH is a tetramer composed of two types of subunits, either H (heart) or M (muscle), the combination of which gives rise to its five isoenzymes found in mammalian tissues (13, 14). Serum LDH is an effective biochemical marker which is useful in the early diagnosis of pre-eclampsia and can reflect the disease’s severity so that appropriate measures can be taken to reduce morbidity and mortality associated with the disease. Several studies reported that serum LDH level increases with severity of preeclampsia, and showed significant correlation with high blood pressure and poor maternal and perinatal outcomes (12, 13, 15, 16). In preeclampsia, multiple systems of the body such as renal, cardiovascular, hematological and nervous system are affected leading to cellular death and consequent leakage of LDH from cells and therefore, its raised level (11).

In order to prevent PE, the disease must be diagnosed at its earliest stage however, the triads of high blood pressure, edema and albuminuria is neither specific nor sensitive enough for diagnosing the disease. Hence, reliable biomarker/s have to be searched for and evaluated to be used as golden diagnostic tools (13, 17). Several potential biochemical markers have been proposed to predict the severity of preeclampsia. Enzymes known as markers of cellular damage are one among others, more specifically, LDH is most useful (4, 18, 19).

In spite of many studies conducted among pregnant women suffering from pregnancy related hypertensive complications like preeclampsia at international level (19–23), there are no studies undertaken in Ethiopia aimed at investigating the role of LDH as a potential biomarker in predicting the onset of preeclampsia at its earlier stage so far. This study aimed to investigate the diagnostic performance of serum LDH, which is cost effective and easy to test, as a potential clinical biomarker for preeclampsia. This study might help clinicians to give due emphasis on employing serum LDH as an independent biomarker for detecting preeclampsia at an earlier and therefore, potentially more treatable stage. In addition, the result of this study might serve as a baseline datum for researchers who are interested in conducting further related studies.

## Materials and methods

### Study design, area and period

We conducted a hospital based cross-sectional study at Debre Birhan Comprehensive Specialized Hospital (DBCSH) as of September 9 to December 24, 2022. DBCSH is a regional tertiary hospital housing four departments namely; medical, surgical, pediatrics and obstetrics and gynecology ward. This study was specifically undertaken in obstetrics and gynecology ward of the hospital.

### Population and study variables

We enrolled total of 132 study subjects, of which preeclamptic and healthy controls each account 66 participants. Among study participants with preeclampsia based on disease’s severity, 33 were severe preeclamptics and the remaining 33 were mild preeclamptics. All pregnant women with confirmed preeclampsia who came for antenatal care (ANC), disease follow up at outpatient department of obstetrics and gynecology ward and those who were hospitalized for better management of the disease during the study period were included in this study. Likewise, apparently healthy age matched pregnant women who came for ANC service, those who were present as attendants in obstetrics and gynecology ward of the hospital at the time of data collection have been included in our study. Both preeclamptic and control groups who get involved in the study were in their second and third trimesters of pregnancy.

On the contrary, pregnant women with pre-existing hypertension or chronic hypertension, gestational or pre-existing diabetes mellitus, thyroid disorder, epilepsy, renal or liver diseases, history of alcoholism and/or smoking, and hemolytic anemia were excluded from the study. Furthermore, we excluded preeclamptics and age matched healthy pregnant women who were the first trimester of pregnancy.

Serum LDH was considered as dependent variable, while disease duration, family and previous history of preeclampsia, gestational and maternal age, anthropometric parameters, and clinical parameters (blood pressure, antihypertensive therapy) were taken as independent variables.

### Data collection and measurement

Once informed consent was taken from each study participant, all necessary information regarding sociodemographic and clinical characteristics were collected using a structured questionnaire by trained data collectors through face-to-face interviews and by reviewing participants’ medical records. Physical measurements like height and weight needed to calculate body mass index (BMI) were taken directly from each participant. Height was measured using a height measuring scale with light clothing and without shoes, and weight was measured using a standard balance. Blood pressure was measured using mercury sphygmomanometer, and the average of two measurements was noted to determine systolic blood pressure (SBP) and diastolic blood pressure (DBP) in each participant. Both blood pressure (SBP and DBP) and serum LDH were measured on the same day of data collection for all study participants.

Three milliliter (mL) of blood was drawn by venipuncture from medial antecubital vein, and poured into a serum separator tube (SST) which was then taken to the clinical chemistry laboratory unit of DBCSH, and centrifuged for 10 min at 3000 rpm and room temperature, within 2 h of withdrawal to obtain cell free serum. The blood sample collected from study subjects was then analyzed for determination of serum LDH using a fully automated, highly-sensitive, quantitative chemistry analyzer called Cobas^©^ 6,000 at clinical chemistry unit of DBCSH by experienced laboratory technologists.

### Statistical data analysis

All Statistical data analyses were done using SPSS version 25.0 and Med-Calc Statistical Software Version 20.118. Categorical data were presented in frequency and percentage. After all data of quantitative variables were checked for normality using the Kolmogorov–Smirnov test, normally distributed variables were displayed as mean ± standard deviation (SD), and non-normally distributed (skewed) continuous variables were expressed as the median and interquartile range (IQR). Independent sample t-test was used for the comparison of variables between preeclamptic patients and age matched healthy pregnant women. Multiple linear regression was employed to figure out factors associated with serum level of LDH. Receiver operating characteristic (ROC) curve was constructed based on the best tradeoff between sensitivity and specificity to assess the diagnostic accuracy of LDH in predicting preeclampsia.

The optimal cutoff point for LDH was obtained based on the maximum Youden’s index (J) = maximum (sensitivity + specificity − 1). The point corresponding to the maximum Youden’s index was considered a cutoff value. Sensitivity and specificity for preeclampsia diagnosis were also measured. The area under the ROC curve (AUC) was calculated to figure out the diagnostic ability of LDH. All statistical tests were two-tailed, and *p*-value <0.05 at 95% confidence interval (CI) was taken as statistically significant.

## Results

### Sociodemographic characteristics

A total of 132 study participants, 66 preeclamptic and 66 healthy controls, were enrolled in this study. The minimum and maximum age (in years) of the participants was 17 and 37, respectively. The mean age of preeclamptic and healthy control groups was 27.4 ± 5.6 and 26.8 ± 6.1, respectively. Regarding educational status of the participants, 77 (58.3%) of them completed primary and secondary school education while 34 (25.8%) of them completed college or university level of education. Concerning occupation, more than a third 54 (40.9%) of the participants were housewives followed by government employees which accounted for 35 (26.5%). Regarding the participants’ place of residence, 94 (71.2%) and 38 (28.8%) of them were urban and rural residents, respectively, (Table 1).

**Table 1:** Sociodemographic profile of study participants at DBCSH, January 2023.

Variable		Study group with total number (N)		
		Preeclampsia (66)	Control(66)	*p*-value
^a^Age (years)		27.4 ± 5.6	26.8 ± 6.1	0.089
^b^Educational level	Primary school	19(28.8)	21 (31.8)	0.06
	Secondary school	17 (25.8)	20 (30.3)	0.10
	College/university	19(28.8)	15 (22.7)	0.051
	Illiterate	11 (16.7)	10 (15.2)	0.078
Occupation	Government employee	17 (25.8)	18 (27.3)	0.23
	Self-employed	17 (25.8)	11 (16.7)	0.054
	Housewife	23 (23.4)	31 (47)	0.062
	Merchant	5 (7.6)	5 (7.6)	0.085
	Student	4 (6.1)	1 (1.5)	0.069
Place of residence	Urban	50 (75.8)	44 (66.7)	0.26
	Rural	16 (24.2)	22 (33.3)	0.091

a Continuous variable expressed in mean ± standard deviation.

b Categorical variables represented in frequency and percentage (in parenthesis).

### Anthropometric and obstetric profiles of study participants

Concerning anthropometric parameters of study participants, mean values of both weight and height were higher in preeclamptic group (68.7 ± 8 and 1.64 ± 0.08 respectively) than healthy controls. Regarding parity status of the study participants, 32 (48.5%) of both preeclamptics and healthy controls were multipara. Likewise, 28 (42.4%) of preeclamptics and 22 (33.3%) of healthy controls were nullipara (Table 2).

**Table 2:** Anthropometric and obstetric profiles of study participants at DBCSH, January 2023.

Variable		Study group with total number(N)		
		Preeclampsia (66)	Control (66)	*p*-value
Weight		68.7 ± 8	62.6 ± 8	0.062
Height		1.64 ± 0.08	1.62 ± 0.09	0.071
Body Mass index (BMI)		25.5 ± 3.4	23.6 ± 2.8	0.08
^a^SBP (mmHg)		150.2 ± 10.8	119.5 ± 19.6	**0.041**
DBP (mmHg)		101.8 ± 9.5	86.1 ± 17.4	**0.002**
^a^Gestational age (weeks)		33.9 ± 4	33.2 ± 3.6	0.083
^b^Parity	Nullipara	28 (42.4)	22 (33.3)	0.092
	Primipara	4 (6.1)	10 (15.2)	0.078
	Multipara	32 (48.5)	32 (48.5)	0.45
	Grand multipara	2 (3)	2 (3.03)	0.45
Gravidity	Primigravida	27 (40.9)	39 (59.1)	0.058
	Multigravidae	39 (59.1)	27 (40.9)	0.052

a Continuous variable expressed in mean ± standard deviation.

b Categorical variables represented in frequency and percentage (in parenthesis).

**Figure 1:**
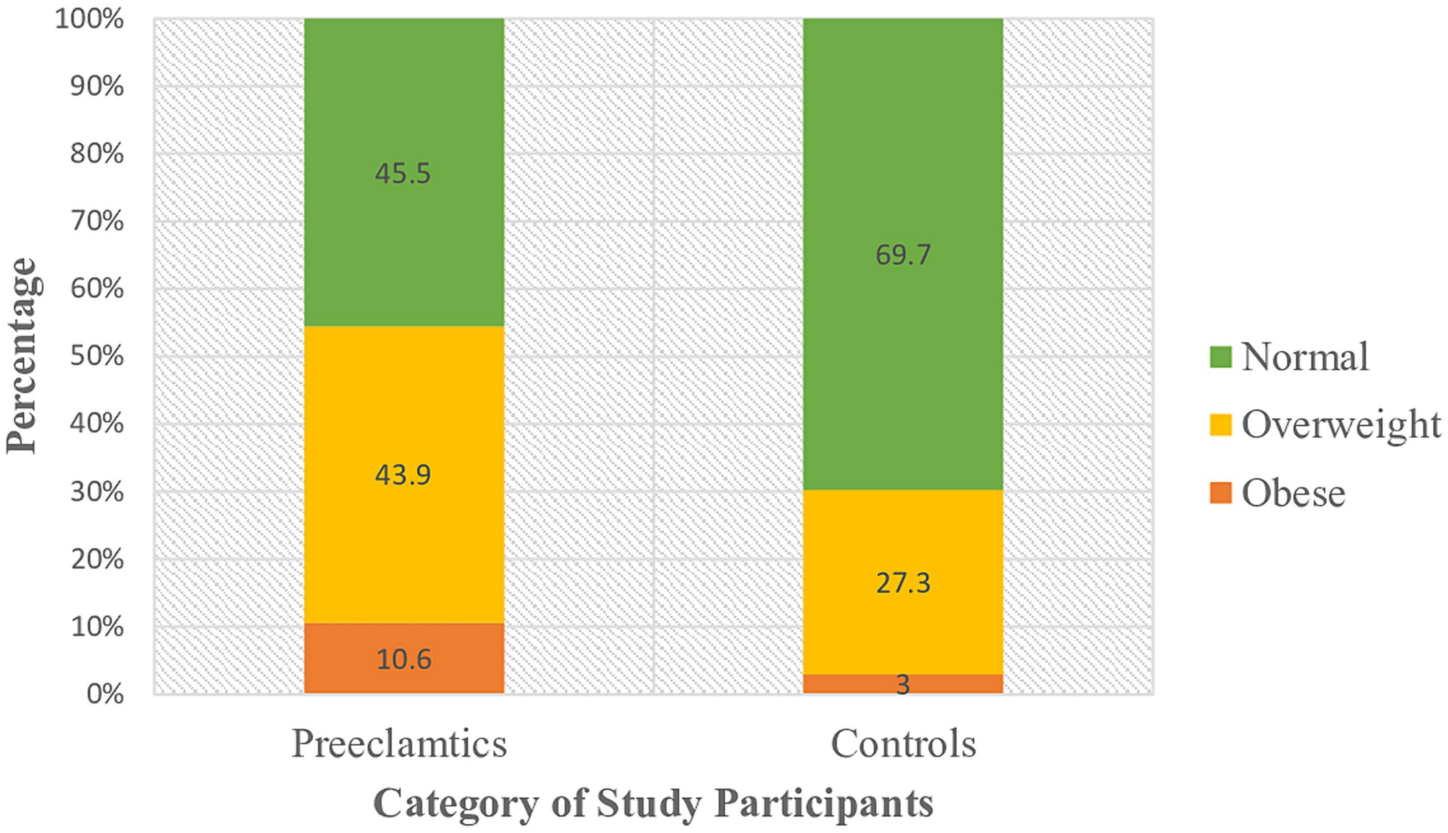
Classification of study participants on the basis of their BMI category at DBCSH, January 2023.

**Figure 2:**
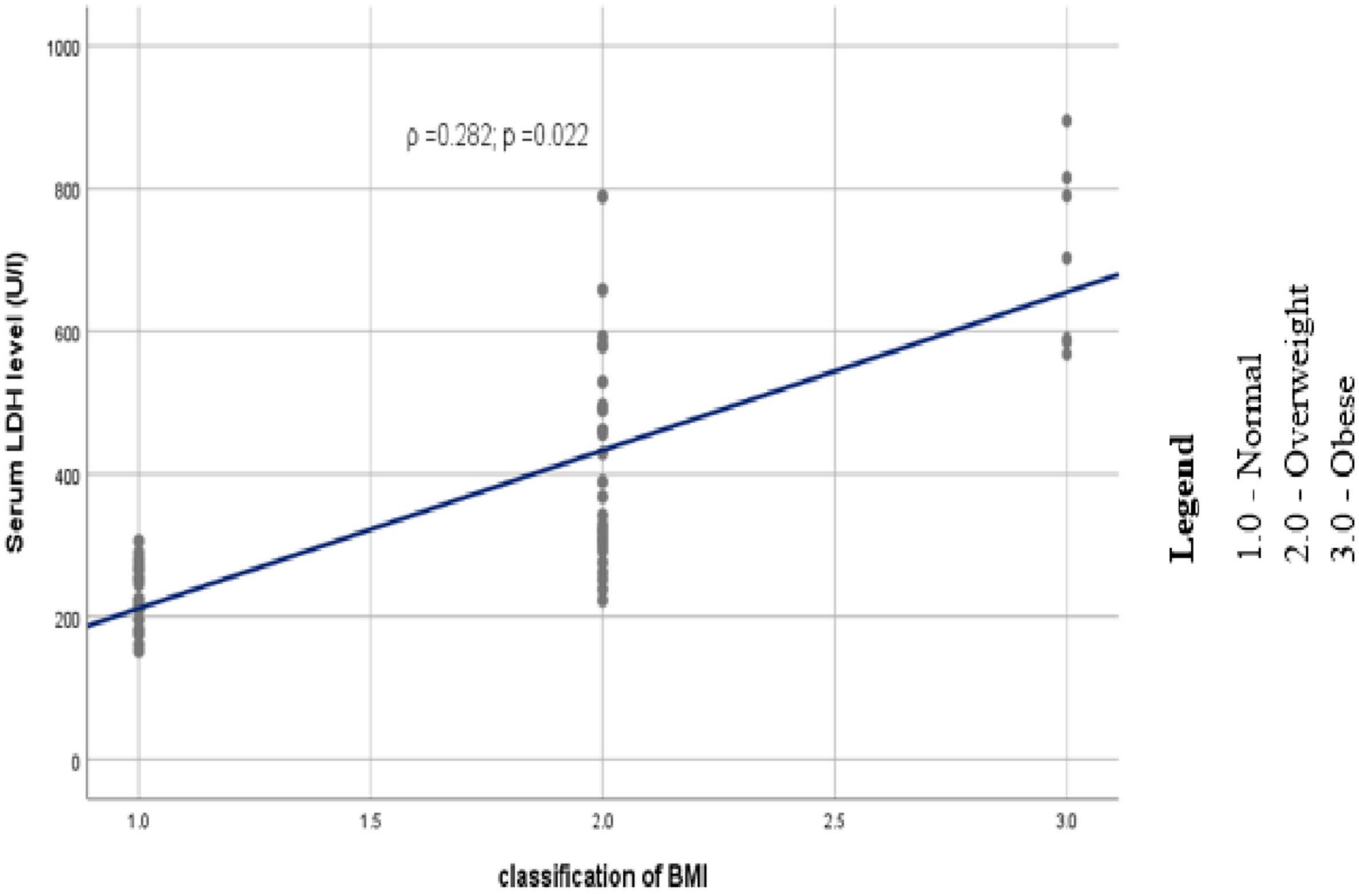
Scatter plot depicting the trend of association between serum LDH and category of BMI among study participants at DBCSH, January 2023.

Study participants were grouped under three classes based on their body mass index. Among preeclamptic study groups, 30(45.5%) were normal, 29 (43.9%) and 7 (10.6%) of them were overweight and obese, respectively. Likewise, 46 (69.7%), 18 (27.3%), and 2 (3%) of the controls were normal, overweight and obese, respectively, (Figure 1). Analysis using Spearman’s correlation revealed that statistically significant positive correlation existed between serum LDH level and BMI category (ρ =0.282; *p* = 0.022), that is as we proceed from normal to overweight and then to obese categories of BMI, serum level of LDH as well showed an increment (Figure 2).

### Clinical profiles of study participants

Twenty-five (37.9%) of study participants from the case group reported family history of preeclampsia. On the other way, 22 (33.3%) of the cases had previous history of preeclampsia. Thirty (45.5%) preeclamptic study participants were using specific antihypertensive drug/s (Table 3).

**Table 3:** Clinical profile of study participants at DBCSH, January 2023.

Variables	Study group with total number (N)	
		Preeclampsia (66)
^a^Disease duration (Days)		28.3 ± 19.8
^b^Family history of Preeclampsia	Yes	25 (37.9)
	No	41 (62.1)
Previous history of Preeclampsia	Yes	22 (33.3)
	No	44 (66.7)
Antihypertensive drug use	Yes	30 (45.5)
	No	36 (54.5)

a Continuous variable expressed as mean ± standard deviation.

b Categorical variables represented in frequency and percentage (in parenthesis).

### Assessment of serum LDH

The normal serum level of LDH ranges from 135 to 214 U/L. Serum level of LDH showed elevation from its acceptable upper limit in more than three-fourth of the cases which is numerically 57 (86.4%). On the contrary, only 5 (7.6%) of healthy controls showed elevated serum LDH level. The remaining study participants’ serum LDH level was in its normal range.

The mean level of LDH in preeclamptic and healthy control study groups was found to be (428.5 ± 25.4 and 170.7 ± 3.8) respectively. This study also showed that there existed statistically significant difference in mean LDH level between preeclamptic and healthy control study groups (*p* < 0.05). Furthermore, our study revealed mean difference of serum LDH level between the two preeclamptic subgroups (mild and severe preeclamptics) to be statistically significant (*p* = 0.008) (Table 4).

**Table 4:** Comparison of serum LDH level of preeclamptic and healthy control study groups, and subgroups of preeclampsia at DBCSH, January 2023.

	Study group with total number (N)					
^a^Serum LDH (U/L)	Preeclampsia	Control	*p*-value	Mild preeclampsia	Severe preeclampsia	*p*-value
	428.5 ± 25.4	170.7 ± 3.8	0.002*	292.3 ± 15.9	457.4 ± 32.6	0.008*

a Values of parameters are expressed in mean ± standard error. *Significant in t-test (two-tailed).

### Diagnostic performance of LDH

**Figure 3:**
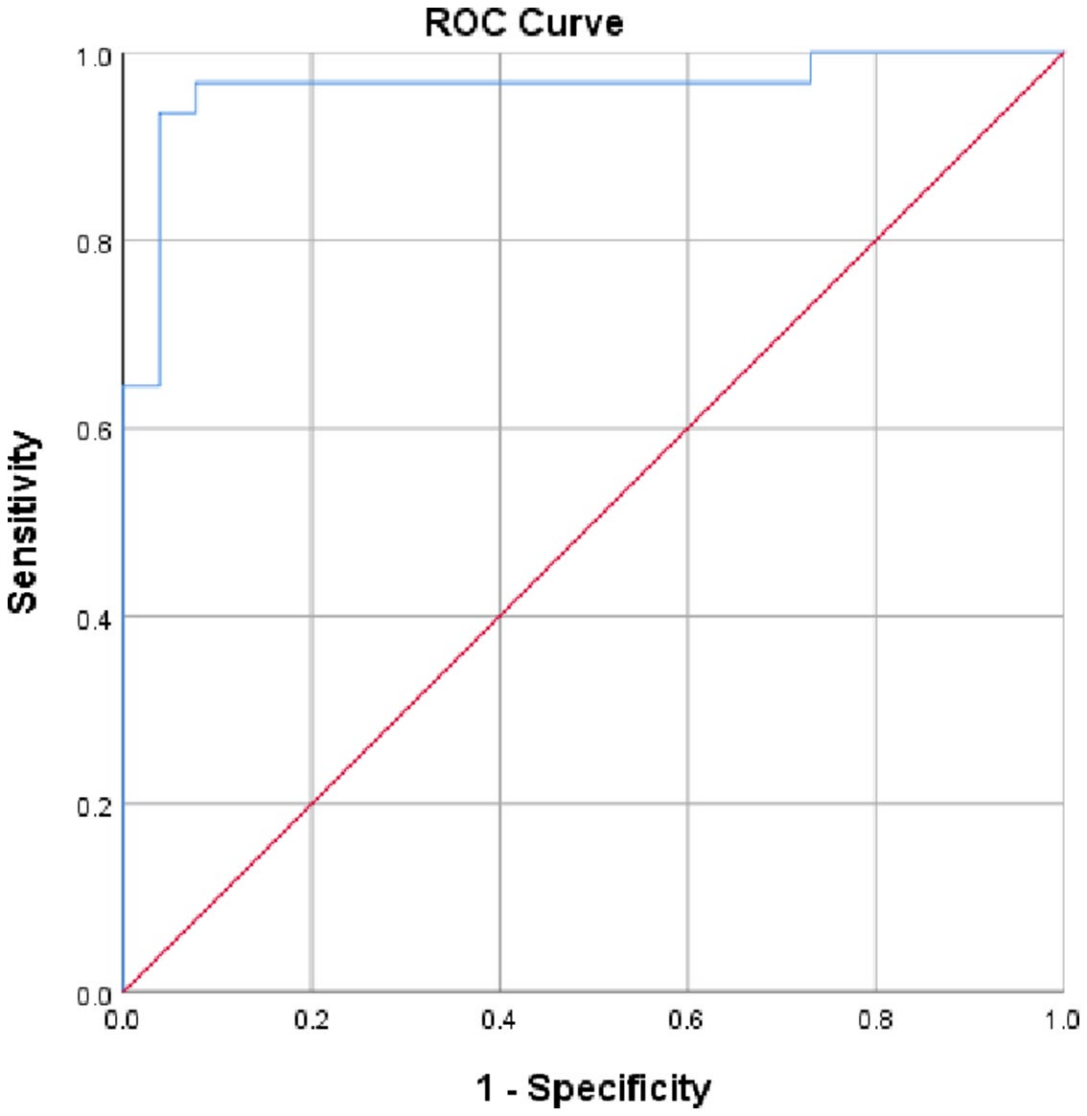
ROC curve revealing diagnostic performance of LDH in identifying preeclampsia at DBCSH, January 2023.

We found the area under the curve (AUC) of LDH, measuring its overall performance, to be 0.963 (95% CI, 0.91, 1.0, *p* = 0.000) from ROC curve analysis. As it can be understood from the ROC curve below, AUC of LDH was significantly different from 0.5, and hence it did show good overall diagnostic performance in identifying preeclampsia from healthy controls. From the ROC curve analysis, the optimal cut-off point for LDH was found to be 376.5 U/L, the sensitivity and specificity of which were 87.5 and 90.8%, respectively, (Figure 3).

### Association of LDH with predictor variables

As it is understood from multiple linear regression analysis given below, systolic blood pressure (SBP), disease duration, gestational age and diastolic blood pressure (DBP) showed independent considerable association with serum LDH level. DBP was found to be a significant positive predictor of serum LDH level among pregnant women with preeclampsia. More specifically, for 1 mmHg increase in DBP, serum LDH level increased by a factor of 4.81 U/L. Likewise, 1 mmHg increase in SBP resulted in elevation of serum LDH by 3.21 U/L. Furthermore, for a one-week increase in gestational age, serum LDH level decreased by 15.39. Lastly, for a one-day increase in duration of disease, serum level of LDH was found to accrue by a factor of 5.32 (Table 5).

**Table 5:** Multiple linear regression depicting factors associated with serum LDH at DBCSH, January 2023.

Serum LDH (U/L)				
Variables		95% CI for B		
	B*	Lower	Upper	*p*-value
Systolic blood pressure	3.21	−2.18	8.66	**0.04**
Diastolic blood pressure	4.81	10.95	1.34	**0.003**
Weight	8.67	−5.93	10.26	0.12
Gestational age	−15.39	−26.59	−4.19	**0.008**
Disease duration	5.32	7.64	3.01	**0.01**
Height	−0.52	−0.536	−0.491	0.23
BMI	−26.78	−20.46	47.86	0.91

B* indicates unstandardized model coefficient; CI: confidence interval; *p*-values shown in bold are significant at < 0.05.

## Discussion

In the present study, the mean level of LDH in preeclamptic and healthy control study groups was found to be 428.5 ± 25.4 and 170.7 ± 3.8, respectively. A study done in India found mean serum level of LDH in preeclamptic study subjects to be 417.84 U/L and 257.24 U/L in healthy controls.

Another research undertaken in India reported mean serum level of LDH to be 513.37 ± 203.44 and 261 ± 78.47 in preeclamptic and healthy controls, respectively. In addition, a study done in Ethiopia reported mean level of serum LDH in preeclamptic study groups to be 353 ± 132.8 which was slightly lower than the finding of the present study (8, 24, 25). This observed discrepancy might possibly be due to the lowering effects of antihypertensive drugs, which were being taken by some of our study subjects. Besides, comorbidities particularly anemia and diabetes mellitus were recorded in the latter Indian research’s study subjects and hence, might significantly account for raised serum LDH level.

This study also showed that there existed statistically significant difference in serum mean LDH level between preeclamptic and healthy control study groups (*p* < 0.05). Our finding is supported by a number of studies (4, 8, 13, 16). Furthermore, systolic and diastolic blood pressure showed independent association with serum LDH level in this study. This finding was consistent with a number of previous studies done on similar subjects (13, 16, 24, 26, 27).

The mean level of serum LDH in preeclamptics of our study who were on specific antihypertensive therapy/s was 392.3 ± 12.7, however, mean level of serum LDH in preeclamptic study participants who were not taking antihypertensive drugs was found to be 448.5 ± 25.9. According to some textbooks, lower mean serum LDH level observed in those who were on antihypertensive therapy, is supposed to be due to decreased tissue damage and disease progression due to the effect/s produced by the drug/s.

In our study, the AUC for serum LDH, measuring its overall performance to predict development of preeclampsia, was 0.963, and its optimal cut-off point was found to be 376.5 U/L having a sensitivity and specificity of 87.5 and 90.8%, respectively. This finding was higher than a research conducted in India on similar subjects which found AUC for serum LDH to be 0.813, with sensitivity and specificity of 83.8 and 58%, respectively, at 250 U/L as cut-off value, and a study done in Iran which reported AUC for serum LDH 0.805 at an optimal cut-off point of 336 U/L with high sensitivity and moderate specificity (89.62 and 59.3% respectively) (15, 25). On the contrary, a study done by Khalil et al. in Saudi Arabia reported sensitivity of LDH to identify severe preeclampsia as 100%. Another research conducted by Duan et al. in China stated the specificity of LDH to be 92.5% both of which had higher finding than our study did (28, 29). This discrepancy might arise from genetic variation of study participants involved and different techniques used for determining serum LDH cut-off values. This difference might be attributed to variations in specimen storage conditions before assay and delay in transportation of blood sample as reported in Wu et al.’s study.

## Conclusion

Serum LDH had an AUC of greater than 0.8 and showed good diagnostic accuracy in predicting development of preeclampsia. Serum LDH had high sensitivity and specificity for tissue damage associated with preeclampsia. Disease duration, gestational age, systolic, and diastolic blood pressure among enormous number of predictor variables had association with serum level of LDH.

## Data availability statement

The raw data supporting the conclusions of this article will be made available by the authors, without undue reservation.

## Ethics statement

The studies involving humans were approved by Jimma university research directorate. The studies were conducted in accordance with the local legislation and institutional requirements. The participants provided their written informed consent to participate in this study.

## Author contributions

All authors of the manuscript did in collaboration for the successful completion of the study and hence we do not have separate role/s to be described for each author. Therefore, the standard statement of contribution provided in this proof is a good descriptive and we all accept it to be the form in the article being published.
